# Graphene Oxide-Modified Titanium Dioxide Nanotubes Promote Schwann Cell Function and Neurotrophic Factor Expression

**DOI:** 10.3390/jfb17050235

**Published:** 2026-05-08

**Authors:** Xu Cao, Caiyun Wang, Ran Lu, Yanting Mu, Jiangqi Hu, Bin Luo, Su Chen

**Affiliations:** Beijing Stomatological Hospital, Capital Medical University, Beijing 100070, China

**Keywords:** titanium dioxide nanotubes, graphene oxide, Schwann cells, osseoperception, dental implants, neurotrophic factors

## Abstract

This study aims to investigate the effects of graphene oxide-modified titanium dioxide nanotube (TNT-GO) coatings on the biological behavior of Schwann cells and to evaluate their potential applications in dental implant surface modification and peripheral nerve regeneration. Titanium dioxide nanotubes (TNTs) were prepared by anodic oxidation, and graphene oxide (GO) was deposited on their surfaces by electrochemical deposition. The surface morphology and physicochemical properties were characterized by scanning electron microscopy (SEM), Raman spectroscopy, atomic force microscopy, X-ray diffraction, and contact angle measurements. The viability, proliferation, and adhesion of Schwann cells were assessed by cell counting kit-8 assay, live/dead staining, and SEM observation. The expression levels of nerve growth factor (NGF) and glial cell line-derived neurotrophic factor (GDNF) were evaluated by immunofluorescence staining and real-time reverse-transcriptase polymerase chain reaction. The results indicated that TNT-GO surface significantly improved surface hydrophilicity and biocompatibility. Compared with the Ti and TNT groups, Schwann cells on TNT-GO surfaces exhibited enhanced proliferation, better spreading morphology, and significantly increased expression levels of NGF and GDNF. Overall, TNT-GO effectively promotes Schwann cell proliferation, adhesion, and neurotrophic factor secretion, suggesting its potential as a novel surface modification strategy to promote peri-implant nerve regeneration and improve osseoperception.

## 1. Introduction

Since Brånemark proposed the concept of osseointegration, dental implant restoration has become an important approach for the replacement of missing teeth, showing high success rates and long-term stability [1,2]. However, several challenges still exist in clinical applications. One of the main issues is the loss of sensory function caused by the absence of the periodontal ligament. In natural teeth, numerous mechanoreceptors within the periodontal ligament can sense occlusal forces and transmit signals to the central nervous system through the trigeminal nerve, enabling precise control of occlusion. Because implants lack the periodontal ligament, normal mechanosensation is lost. As a result, excessive occlusal loading is difficult to detect in time, which may lead to screw loosening, prosthesis fracture, or even failure of osseointegration. The loss of sensory feedback not only increases the risk of mechanical complications but also makes occlusal adjustment more difficult for clinicians [3,4]. Rebuilding the neural feedback pathway around implants and restoring or enhancing osseoperception has become a research focus in dental implantology.

The concept of osseoperception was first proposed in the 1990s and has received increasing attention. Studies have found that even without the periodontal ligament, patients can still perceive implant load and identify occlusal contact to some extent. This suggests that nerve remodeling and feedback pathways may still exist around the implants [5,6]. Schwann cells play a key role in peripheral nerve regeneration. They are primary glial cells of the peripheral nervous system that can dedifferentiate and proliferate after nerve injury, forming Büngner bands that guide axonal regeneration [7]. Schwann cells also secrete neurotrophic factors such as nerve growth factor (NGF) and glial cell line-derived neurotrophic factor (GDNF), which are essential for neuron survival, axonal extension, and myelination [8]. Therefore, activating and promoting Schwann cell function is a key step in achieving neural reconstruction and restoring osseoperception around implants.

Currently, strategies to promote peri-implant nerve regeneration mainly include constructing biomimetic periodontal ligament-like structures, Schwann cell transplantation, application of mesenchymal stem cells, and delivery of exogenous growth factors [9,10,11,12]. These methods have shown positive effects in experimental studies but still face challenges such as limited stability, immune rejection, and complex clinical procedures. In contrast, surface modification of implants is a simple and direct approach. It can modulate cell adhesion, proliferation, and differentiation by altering surface morphology, chemical composition, and physical properties. This can improve the interaction between the implant and surrounding bone, soft tissue, and nerve systems [13,14].

Titanium dioxide nanotubes (TNTs) are one of the most widely studied surface modification materials in recent years. TNT is prepared by anodic oxidation and possesses a highly ordered nanotube array structure with a large surface area and tunable morphology [15]. Studies have shown that TNT can enhance osteoblast mineralization, promote fibroblast and epithelial cell adhesion, and have certain anti-inflammatory effects [16,17,18]. In neural repair studies, TNT has also been shown to promote Schwann cell proliferation and the secretion of neurotrophic factors [13]. In addition, their nanotubular structure can serve as a carrier for drugs or bioactive molecules, forming a local sustained-release system to further enhance biological effects [19].

Graphene oxide (GO) is a novel two-dimensional carbon-based nanomaterial that has attracted great attention in the field of nerve regeneration. It contains carboxyl, hydroxyl, and epoxy groups, exhibiting excellent hydrophilicity and biological activity. Many studies have shown that GO can regulate neuronal adhesion, migration, and differentiation, promote axonal growth, and upregulate the expression of neurotrophic factors [20,21]. In Schwann cell-related studies, GO has been reported to enhance their proliferation, migration, and myelination capabilities [22]. Furthermore, GO has antibacterial, anti-inflammatory, and biocompatible properties, which provide additional advantages for application in dental implant modification [23].

However, few studies have systematically investigated the role of graphene oxide-modified titanium dioxide nanotube (TNT-GO) composite coatings in regulating Schwann cell behavior and improving implant osseoperception. Therefore, this study prepared a TNT-GO composite coating using a combination of anodic oxidation and electrochemical deposition. The physicochemical properties of the coatings were characterized, and its effects on Schwann cell adhesion, proliferation, and neurotrophic factor expression were evaluated in vitro. We hypothesize that the incorporation of GO can further enhance the effects of TNT on Schwann cells, providing experimental evidence for a new surface modification strategy to promote neural regeneration and restore osseoperception around dental implants.

## 2. Materials and Methods

### 2.1. Material Preparation

(1) Preparation of TNT

Commercially titanium sheets (10 mm × 10 mm × 0.2 mm; 99.99% purity; Cuibolin Nonferrous Metal Industry Co., Ltd., Beijing, China) were used as substrates. The samples were ultrasonically cleaned in acetone, absolute ethanol, and deionized water for 10 min each to remove surface oil and impurities. The cleaned titanium sheets were used as the anode and a platinum sheet as the cathode. Anodization was performed in an ethylene glycol-based electrolyte containing 0.5 wt% NH_4_F and 10 vol% H_2_O at a constant voltage of 50 V for 15 min. After anodization, the samples were thoroughly rinsed with deionized water and dried at room temperature. To improve crystallinity, the samples were annealed in a muffle furnace at 550 °C for 2 h, thereby transforming the amorphous oxide layer into crystalline anatase TiO_2_ nanotubes.

(2) Preparation of TNT–GO

The prepared TNT samples were immersed in an aqueous graphene oxide (GO, Sigma–Aldrich, St. Louis, MO, USA) solution at a concentration of 0.1 mg/mL. Before electrodeposition, the GO dispersion was ultrasonically treated for 2 h to improve dispersion stability. Electrodeposition was then carried out with TNT as the cathode and a platinum sheet as the anode under a constant voltage of 50 V for 5 min to achieve uniform loading of GO onto the nanotube surface. After electrodeposition, the samples were rinsed with deionized water and dried at room temperature. This protocol was selected based on our previous TNT-GO surface modification study, in which the same GO loading strategy was shown to introduce oxygen-containing functional groups onto TNT surfaces and improve the biological performance of titanium-based substrates [18].

Finally, three types of samples were obtained for subsequent experiments: Ti group: a polished titanium surface; TNT group: an anodized titanium dioxide nanotubes surface; and TNT–GO group: a graphene oxide-modified titanium dioxide nanotubes surface.

### 2.2. Material Characterization

The surface morphology of the samples was observed using a scanning electron microscope (SEM, S4800, Hitachi Ltd., Tokyo, Japan). Raman spectra were recorded using a LabRAM HR800 spectrometer (HORIBA, Paris, France) with a 532 nm laser excitation source, the range of 1000–3000 cm^−1^ was used to identify the D and G bands of GO. Surface roughness was analyzed using an atomic force microscope (AFM, Nanoscope V, Veeco, Santa Barbara, CA, USA). The water contact angle (WCA) was measured using an optical contact angle goniometer (Dataphysics OCA20, Filderstadt, Germany) to evaluate surface wettability. The crystal phase of the samples was identified by X-ray diffraction (XRD, TTRAX III, Rigaku Co., Tokyo, Japan).

### 2.3. Cell Culture

The commercial Rat Schwann cells (RSC96, ATCC, China Cell Bank, Beijing, China) were used in this study. Cells were cultured in high-glucose Dulbecco’s Modified Eagle Medium (DMEM, Gibco, Thermo Fisher Scientific, Waltham, MA, USA) supplemented with 10% fetal bovine serum (FBS, Gibco, Thermo Fisher Scientific, Waltham, MA, USA) and 1% penicillin–streptomycin (Sigma-Aldrich, St. Louis, MO, USA). All cells were maintained at 37 °C in a humidified atmosphere with 5% CO_2_. Cells between passages 3 and 6 in the logarithmic growth phase were used for experiments.

### 2.4. Biological Performance Evaluation

(1) Hemolysis Test

A 0.5 mL volume of blood was collected from the orbital vein of healthy SD rats. Blood was centrifuged at 5000 rpm for 10 min at 4 °C to remove serum, and red blood cells were retained. Cells were washed five times with phosphate-buffered saline (PBS) to prepare a 2% red blood cell suspension. Ti, TNT, and TNT-GO samples were placed in centrifuge tubes and mixed with 1 mL red blood cell suspension. PBS was used as a negative control, and Triton X-100 as a positive control. After incubation at 37 °C for 3 h, samples were centrifuged, and the absorbance of the supernatant was measured at 450 nm. Hemolysis rate was calculated as: Hemolysis (%) = (Sample OD − Negative control OD)/(Positive control OD − Negative control OD) × 100%. A hemolysis rate below 5% was considered non-hemolytic.

(2) Cell Counting Kit-8 Assay (CCK-8 Assay)

Samples were placed in 24-well plates, and Schwann cells were seeded at 1 × 10^5^ cells/cm^2^ per well. Cells were cultured for 1, 4, and 7 days. At each time point, 10% CCK-8 working solution (CCK-8, Dojindo, Kumamoto, Japan) was added and incubated at 37 °C for 1 h. The supernatant was transferred to 96-well plates, and absorbance at 450 nm was measured to assess cell viability and proliferation.

(3) Cell Morphology and Adhesion

Schwann cells were cultured on the sample surfaces for 24 h and 48 h. Samples were washed with PBS, fixed with 3% glutaraldehyde for 2 h, followed by 1% osmium tetroxide for 1 h. After dehydration through a graded ethanol series (30–100%), samples were vacuum dried, sputter-coated with gold, and observed by SEM to examine cell morphology, spreading, and filopodia formation.

(4) Live/Dead Cell Staining

Cells cultured on the sample surfaces for 24 h and 48 h were washed with PBS and stained with a Calcein-AM/PI working solution (Beyotime Biotechnology, Shanghai, China) at 37 °C for 30 min in the dark. Live cells emitted green fluorescence, while dead cells emitted red fluorescence. Fluorescence microscopy (Olympus IX73, Olympus, Tokyo, Japan) was used for imaging. For quantitative analysis, three randomly selected fluorescence images from each group were analyzed. Calcein-AM-positive green cells were counted as live cells, and PI-positive red cells were counted as dead cells. The live cell ratio was calculated as live cells/total cells × 100%.

(5) Immunofluorescence Staining

After 3 days of culture on the samples, cells were washed with PBS, fixed with 4% paraformaldehyde for 30 min, and permeabilized with 1% Triton X-100 for 10 min. Cells were blocked with goat serum at room temperature for 30 min, incubated with primary antibodies (anti-NGF, 1:200; anti-GDNF, 1:200, ABclonal Technology Co., Ltd., Wuhan, China) at 4 °C overnight. After washing with PBS, cells were incubated with Alexa Fluor 488 secondary antibody (1:500) at room temperature for 1 h. The cytoskeleton was stained with phalloidin for 30 min, and nuclei with 4′,6-diamidino-2-phenylindole (DAPI, Beijing Zhongshan Golden Bridge Biotechnology Co., Ltd., Beijing, China) for 5 min. Fluorescence microscopy was used to observe cell morphology and neurotrophic factor expression.

(6) Gene Expression Analysis (real-time reverse-transcriptase polymerase chain reaction, RT-PCR)

Schwann cells were cultured on samples for 3 days. Total RNA was extracted using Trizol reagent (Invitrogen, Thermo Fisher Scientific, Waltham, MA, USA) and quantified using a Nanodrop 2000 spectrophotometer. A 1 μg quantity of RNA was reverse-transcribed into cDNA. RT-PCR was performed using SYBR Green reagent (Takara Bio Inc., Shiga, Japan) with the following program: 95 °C for 30 s, followed by 40 cycles of 95 °C for 5 s and 60 °C for 30 s. Relative gene expression was calculated using the 2^−ΔΔCt^ method. The primer sequences for the target genes are listed in Table 1.

### 2.5. Statistical Analysis

All data are expressed as mean ± standard deviation (mean ± SD). Statistical analysis was performed using SPSS 26.0. One-way ANOVA was used for comparisons among groups. Differences were considered statistically significant at *p* < 0.05.

## 3. Results

### 3.1. Material Characterization

(1) Surface Morphology (SEM)

**Figure 1:**
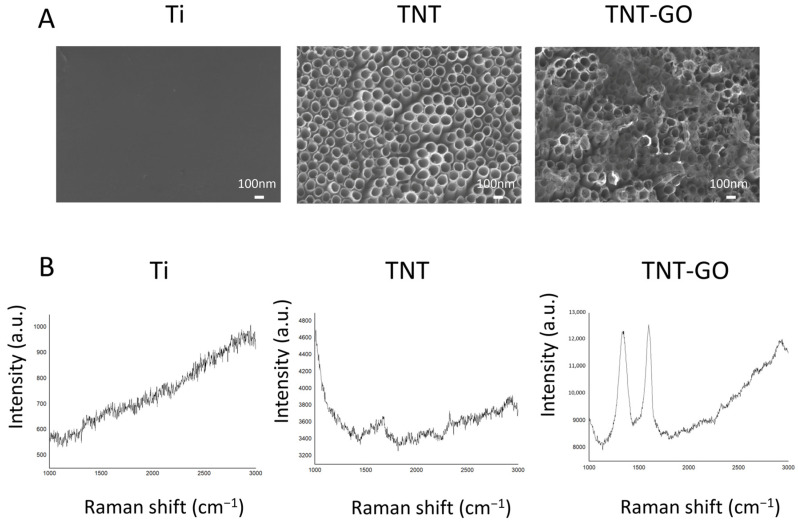
Results of surface characterization of the Ti, TNT, and TNT-GO samples. (**A**) SEM images; (**B**) Raman spectra collected in the range of 1000–3000 cm^−1^ for identifying the characteristic D and G bands of GO. Abbreviations: Ti, titanium; TNT, TiO_2_ nanotubes; TNT−GO, TiO_2_ nanotubes with graphene oxide.

SEM images (Figure 1A) showed that the Ti group surface was relatively smooth, with no obvious nanostructures. The TNT group formed an orderly array of nanotubes with an average diameter of approximately 100 nm, exhibiting a uniform and continuous distribution. In the TNT-GO group, the nanotube surface was covered with wrinkled graphene oxide flakes, forming a layered structure tightly bonded to the TNT substrate, indicating successful deposition of GO on the nanotube surface. To better evaluate the distribution of GO, a high-magnification SEM image at 100,000× was added (Figure S1). The image showed that GO sheets were broadly distributed over the TNT surface and partially covered the nanotube openings. SEM image analysis showed that GO covered 88.59% of the analyzed surface area.

(2) Raman Spectroscopy

Raman spectra in the GO-sensitive range (Figure 1B) showed that the TNT-GO group had obvious D (1350 cm^−1^) and G (1580 cm^−1^) peaks, corresponding to the defect peak and graphitic peak of GO, respectively. No such characteristic peaks were observed in the Ti and TNT groups. This result confirmed the successful loading of GO on the TNT surface. The Raman range used in this study was selected for GO identification rather than for evaluating the characteristic Raman modes of TiO_2_. The crystal phase of the TNT layer was mainly determined by XRD in the present study.

(3) Surface Roughness (AFM)

**Figure 2:**
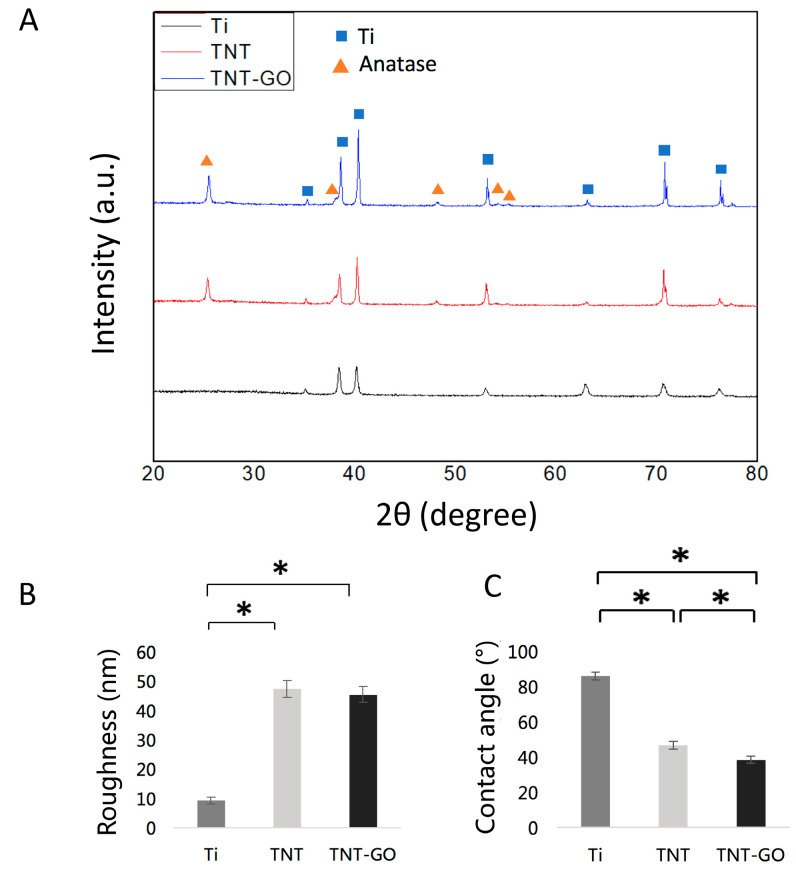
Surface analysis of the Ti, TNT and TNT-GO samples. (**A**) XRD patterns; (**B**) roughness of samples; (**C**) water contact angles. Abbreviations: XRD, X-ray diffraction. * *p* < 0.05.

AFM analysis (Figure 2B) showed that the surface roughness (Ra) of the Ti group was 9.1 ± 1.2 nm, significantly lower than that of both the TNT group (47.2 ± 3.0 nm) and TNT-GO group (45.3 ± 2.7 nm) (*p* < 0.05). There was no statistically significant difference between the TNT and TNT-GO groups (*p* > 0.05).

(4) X-ray Diffraction (XRD)

XRD patterns (Figure 2A) showed that only titanium substrate peaks were present in the Ti group. After heat treatment, the TNT group showed characteristic peaks of anatase TiO_2_, indicating that the oxide layer transformed from an amorphous state to a crystalline state. The TNT-GO group showed the same diffraction peaks as TNT, indicating that GO modification did not alter the crystal structure of the TNT.

(5) Wettability

WCA measurements (Figure 2C) showed that the Ti group had the largest WCA (86 ± 2.4°), indicating that it was a hydrophobic surface. The WCA of the TNT group was significantly reduced (46.6 ± 2.2°), indicating hydrophilicity, while the WCA of the TNT-GO group was even smaller (38.5 ± 1.9°), indicating that GO modification significantly enhanced the surface hydrophilicity.

### 3.2. Hemolysis Test

**Figure 3:**
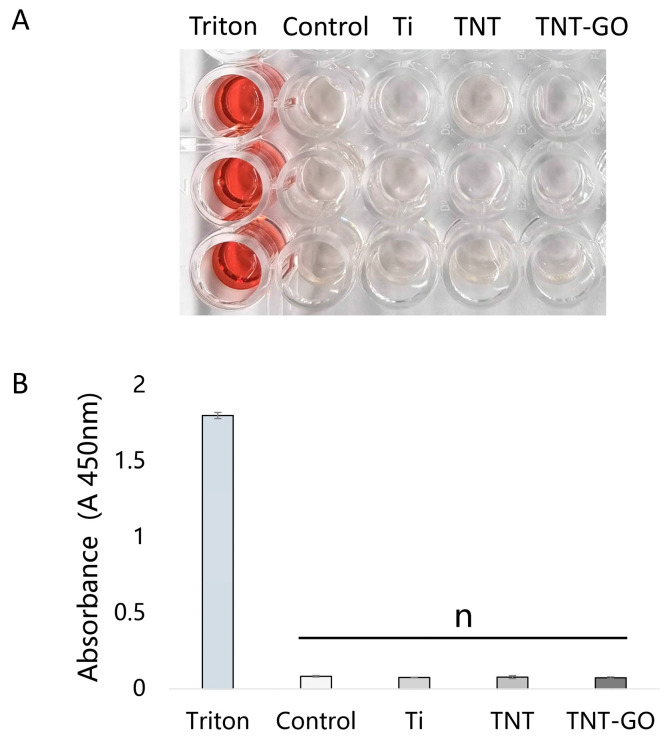
Hemolysis test of Schwann cells on the samples. (**A**) Representative photographs; (**B**) The absorbance values at 450 nm. “*n*” means no statistical difference.

The hemolysis rates (Figure 3) in all three groups were below 5%, indicating no significant hemolysis. The hemolysis rates of the Ti, TNT, and TNT-GO groups were all close to zero.There was no significant difference compared to the negative control group (*p* > 0.05). These results indicated that all three materials had good blood compatibility.

### 3.3. Cell Proliferation (CCK-8)

**Figure 4:**
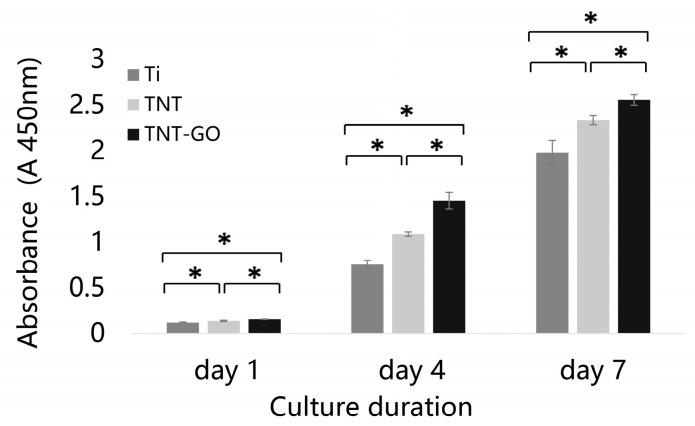
Cell proliferation of Schwann cells on the samples after 1, 4, and 7 days. * *p* < 0.05.

The CCK-8 assay results (Figure 4) showed that Schwann cells proliferated normally on all three sample surfaces, and the cell numbers increasing over time. On days 1, 4, and 7, the TNT-GO group showed the strongest proliferation ability, significantly higher than the TNT and Ti groups (*p* < 0.05). The TNT group also showed significantly higher proliferation ability than Ti (*p* < 0.05). These results indicated that GO modification further enhanced the ability of TNT to promote Schwann cell proliferation.

### 3.4. Cell Morphology and Adhesion (SEM Observation)

**Figure 5:**
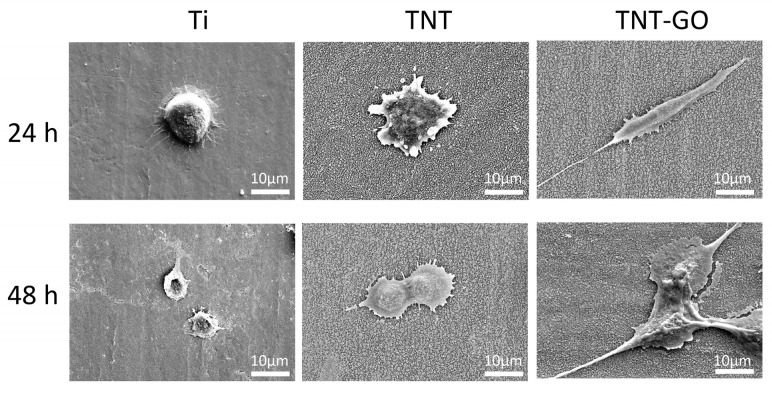
SEM images of Schwann cells on the different surfaces at 24 and 48 h.

SEM images (Figure 5) showed that after 24 h, cells on the Ti group were mostly round with small spreading area and protruding nuclei. In the TNT group, some cells were polygonal with visible filopodia extension. Cells in the TNT-GO group exhibited spindle-shaped morphology with full spreading and long filopodia were tightly attached to the substrate. After 48 h, cells in the Ti group remained round with limited spreading. Cells in the TNT group showed increased polygonal spreading and more filopodia adhesion. Cells in the TNT-GO group were more elongated, with intercellular filopodia formed a network-like structure, and enhanced spreading and adhesion.

### 3.5. Live/Dead Cell Staining

**Figure 6:**
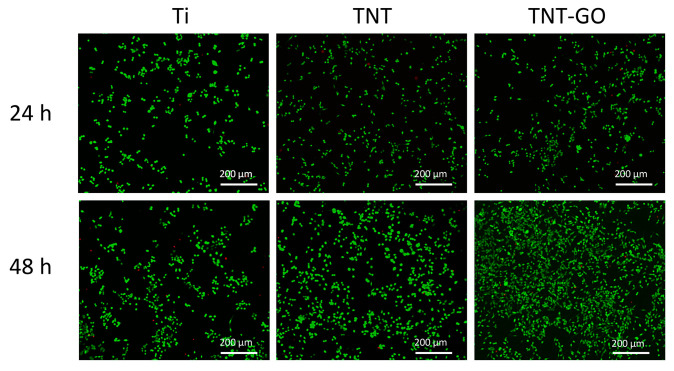
The live/dead viability of Schwann cells at 24 and 48 h.

Calcein-AM/PI staining results (Figure 6) showed a large number of green fluorescent live cells and a small number of red dead cells on the surface of all three samples. The live cell ratios in the Ti, TNT, and TNT-GO groups were 98.1%, 99.1%, and 98.4% at 24 h, and 96.6%, 97.3%, and 97.9% at 48 h, respectively (Figure S2). The proliferation numbers of cells in both the TNT and TNT-GO groups were higher than those in the Ti group. The TNT and TNT-GO groups showed higher cell viability compared with Ti.

### 3.6. Neurotrophic Factor Expression (Immunofluorescence)

**Figure 7:**
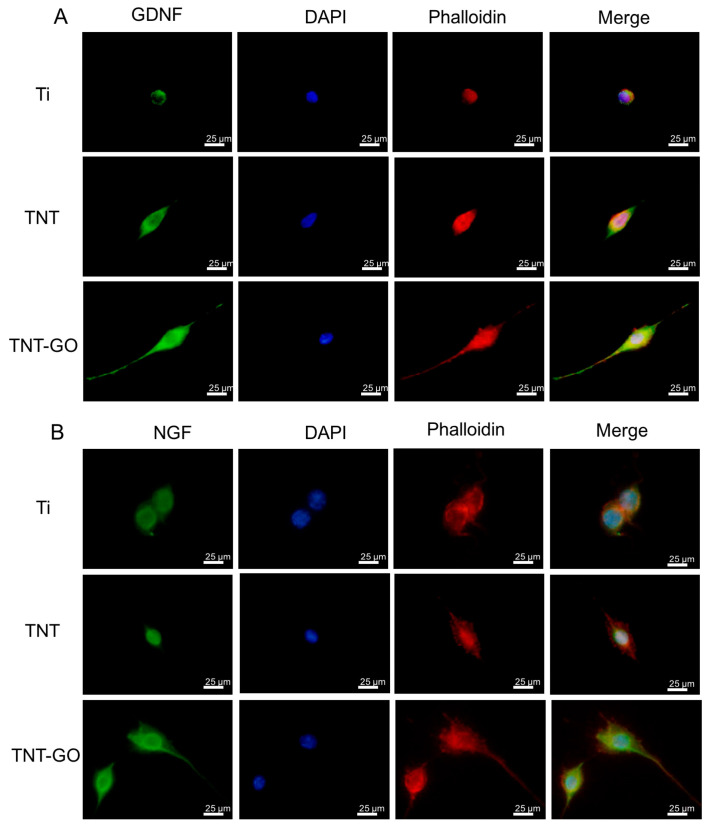
Immunofluorescent staining of Schwann cells at 3 days. (**A**) GDNF was labeled green, and nuclei were stained with DAPI. (**B**) NGF was labeled green, and nuclei were stained with DAPI. ‘Merge’ represents the merged images of GDNF or NGF with Phalloidin and nuclei. Abbreviations: DAPI, 4′,6-diamidino-2-phenylindole. GDNF, Glial cell line-derived neurotrophic factor. NGF, Nerve growth factor.

**Figure 8:**
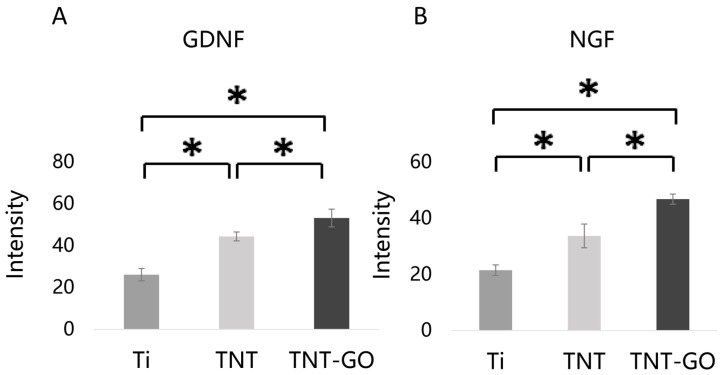
Quantitative analysis of fluorescent staining shown in Figure 7. (**A**) GDNF. (**B**) NGF. * *p* < 0.05.

Immunofluorescence staining (Figure 7) showed that after 3 days of culture, NGF and GDNF protein signals in Schwann cells were stronger in the TNT and TNT-GO groups than those in the Ti group, with the TNT-GO group showing the most significant expression. Quantitative fluorescence analysis (Figure 8) confirmed that NGF and GDNF protein levels in the TNT-GO group were significantly higher than those in the TNT and Ti groups (*p* < 0.05), and the TNT group was significantly higher than Ti (*p* < 0.05).

### 3.7. Gene Expression Levels (RT-PCR)

**Figure 9:**
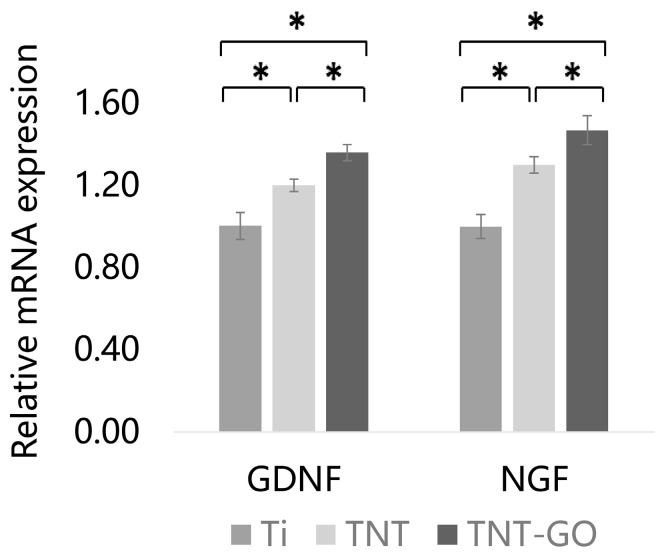
Relative mRNA expression of NGF and GDNF at 3 days. * *p* < 0.05.

RT-PCR results (Figure 9) showed that after 3 days of culture, Schwann cells on the TNT and TNT-GO groups exhibited significantly higher mRNA levels of NGF and GDNF compared with those in the Ti group (*p* < 0.05). The TNT-GO group showed the highest gene expression levels, significantly higher than the TNT group (*p* < 0.05). These results indicated that GO modification further enhanced the promoting effect of TNT on the expression of neurotrophic factor genes in Schwann cells.

## 4. Discussion

This study systematically evaluated the effects of TNT-GO on the biological functions of Schwann cells. The results showed that the composite coating not only significantly improved the surface properties of the material, but also promoted the adhesion, proliferation and expression of neurotrophic factors of Schwann cells. These findings provide a novel approach for functionalization of implant surfaces from the perspectives of materials science and cell biology.

In this study, the materials were divided into three groups: Ti, TNT, and TNT-GO. The Ti used in this study refers to a polished commercially pure titanium substrate with a naturally formed native oxide layer. Titanium can spontaneously form a thin passive oxide layer after exposure to air or aqueous environments [24]. Compared with the anodized TNT surface, the native oxide layer on polished Ti is much thinner and lacks an ordered nanotubular structure. After anodization, a uniform and ordered titanium dioxide nanotube structure is formed. Annealing transformed the TiO_2_ nanotube layer from an amorphous phase to crystalline anatase. The anatase TiO_2_ is generally more stable and has better corrosion resistance and biocompatibility than amorphous TiO_2_ [25]. The formation of nanotube arrays also increased surface roughness compared with polished Ti. Increased roughness can enlarge the surface area and provide nanoscale cues for protein adsorption and cell attachment [18,25]. However, roughness alone cannot explain the enhanced Schwann cell response, because the TNT and TNT-GO groups showed similar roughness values. Therefore, the key differences among the Ti, TNT, and TNT-GO groups in this study are mainly associated with the formation of an ordered TiO_2_ nanotube layer, the crystallization of anatase TiO_2_ after annealing, changes in wettability and nanoscale surface topography, and the introduction of GO-related oxygen-containing surface chemistry.

The surface morphology and physicochemical properties of materials are important factors affecting cell behavior [26]. SEM images showed that TNT formed an ordered array of nanotubes, while GO modification created wrinkled flakes on the surface. In this study, Raman spectroscopy was used to confirm the successful loading of GO on the TNT surface. The characteristic D and G bands at approximately 1350 and 1580 cm^−1^ were detected in the TNT-GO group, indicating the presence of GO. However, this Raman shift range is mainly suitable for GO identification rather than TiO_2_ phase analysis. The characteristic Raman modes of anatase TiO_2_ are mainly located in the low-wavenumber region. Therefore, the TiO_2_ crystal phase in this study was mainly evaluated by XRD. Kim et al. also showed that Raman characterization of titanate/TiO_2_ nanotubes should be performed in the low-wavenumber region below 1000 cm^−1^ [27]. In our previous study using the same TNT system, low-wavenumber Raman spectra showed typical anatase TiO_2_ peaks at approximately 144, 196, 396, 515, and 637 cm^−1^, supporting the formation of anatase TiO_2_ nanotubes under similar conditions [28].

In our previous study using the same TNT-GO system, XPS confirmed GO loading on the TNT surface [18]. The C 1s spectrum showed C–C/C–H, C–OH, C=O/C–O, and O=C–O peaks. The O 1s spectrum showed Ti–O, Ti–OH/C=O, C–O, and C–OH peaks. These results indicated the presence of oxygen-containing surface groups. The TNT-GO surface also showed higher protein adsorption. No obvious coating delamination was found in the nanoscratch test [18]. These findings support the successful preparation and stability of the TNT-GO surface.

The nanoscale structure of TNT can mimic the extracellular matrix (ECM) microenvironment and enhance cell adhesion [29]. After modification with GO, its unique two-dimensional wrinkled structure and abundant oxygen-containing functional groups increased surface energy and protein adsorption, providing more binding sites for cells [18,30]. GO usually contains hydroxyl, carboxyl, epoxy, and carbonyl groups. The amount and type of these groups depend on the preparation method of GO [31]. These functional groups may promote protein adsorption and early cell attachment [32]. At the same time, strong interactions between proteins and highly oxidized GO surfaces may also change protein conformation. In some cases, this may lead to partial protein denaturation [33,34]. The relationship between contact angle, surface hydrophilicity, and cellular behavior is a complex and multifaceted area of research in biomaterials science and tissue engineering. Ferrari et al. also showed that surface properties, including hydrophilicity, can influence mammalian cell adhesion, proliferation, and morphology, but the effect is not simply dependent on maximum hydrophilicity [35]. Optimal cell adhesion is often observed on surfaces with moderate hydrophilicity.

In this study, TNT exhibited a lower WCA than Ti because the anodized TiO_2_ nanotubular layer provides a high-surface-energy oxide surface, larger surface area, hydroxylated sites and open tubular channels that facilitate capillary water penetration [36]. Thus, the TNT surface is more consistent with a Wenzel-like wetting state rather than a Cassie-Baxter “lotus-leaf” state, which usually requires low-surface-energy chemistry and trapped air pockets [37,38]. GO further reduced WCA because its oxygen-containing groups can increase surface hydration. However, the favorable Schwann cell response on TNT-GO may result from the combined effects of nanotube morphology, GO surface chemistry, wettability, protein adsorption, and coating stability, rather than hydrophilicity alone [18].

CCK-8, SEM, and live/dead staining showed that TNT significantly enhanced Schwann cell activity compared with Ti, and TNT-GO had the most significant effect (Figure 4, Figure 5 and Figure 6). This effect may be associated with the integrated surface microenvironment created by TNT-GO, including the ordered nanotube structure, GO-related oxygen-containing groups, improved wettability, increased protein adsorption, and stable coating attachment. These surface features may promote early cell adhesion and spreading, thereby supporting Schwann cell proliferation. Schwann cells are key for peripheral nerve regeneration, and their enhanced activity promotes axon extension and myelin regeneration. Previous studies reported that GO can enhance cell activity, regulate cytoskeleton organization and focal adhesion formation, thereby activating downstream signaling pathways and enhancing cell adhesion and migration [39]. In this study, Schwann cells on TNT-GO exhibited spindle-shaped morphology, extended filopodia, and formed intercellular connections. These changes indicate better cell spreading and adaptation to the TNT-GO surface. Based on the physicochemical characteristics of TNT-GO, this response may be related to the combined effects of ordered nanotube topography, increased surface area, improved wettability, GO-related oxygen-containing groups, and enhanced protein adsorption. These surface features may promote early cell–substrate interactions and then influence cytoskeletal organization and cell spreading. However, these observations are mainly morphological evidence, and the exact molecular mechanisms require further investigation.

Immunofluorescence and RT-PCR results showed that TNT-GO significantly upregulated the expression of NGF and GDNF. These neurotrophic factors play a crucial role in peripheral nerve regeneration and functional recovery. NGF promotes sensory neuron survival and axon growth; GDNF mainly supports motor neurons and enhances anti-apoptotic capacity [40]. Studies have shown that GO can interact with integrin receptors on the cell membrane to regulate downstream signaling, and promote the secretion of neurotrophic factors [20]. Based on these results, TNT-GO may enhance the expression of neurotrophic factors by improving cell adhesion and signaling transduction, thereby supporting neural remodeling around the implant. Nevertheless, the biological effects of GO depend on its concentration, oxidation degree, lateral size, dispersion state, and immobilization form. Although oxygen-containing groups may improve hydrophilicity and protein adsorption, excessive surface oxidation or strong electrostatic interactions may alter protein conformation and induce adverse cellular responses [32,41]. Therefore, the positive Schwann cell response observed in this study should be interpreted within the low-concentration, surface-immobilized TNT-GO coating system. It should not be directly generalized to high-dose or dispersed GO exposure.

Previous studies mainly focused on the effects of TNT on osteoblasts or fibroblasts, with limited reports on nerve repair. Few studies have suggested that TNT can promote Schwann cell adhesion, but systematic functional validation is lacking [13]. GO has been applied in neural tissue engineering, such as promoting neural stem cell differentiation or axon regeneration in peripheral nerve injury models [42,43]. This study is the first to combine TNT with GO and validate its effects on Schwann cell biological functions, proposing TNT-GO as a potential strategy for promoting nerve reconstruction around implants. This provides experimental support for the concept of “neuro-integrated implants”.

Implants lacking periodontal ligament and adequate occlusal sensation, often leading to excessive occlusal forces and mechanical complications. The concept of “osseoperception” highlights the importance of nerve reconstruction around implants for sensory recovery. This study suggests that TNT-GO can support peripheral nerve fiber regeneration by promoting Schwann cell function, potentially restoring part of the sensory function. Furthermore, GO possesses antibacterial and anti-inflammatory properties, which may help prevent peri-implantitis [44]. Therefore, TNT-GO coatings have multidimensional potential to improve long-term stability of implants.

Despite these promising results, this study has limitations. First, the experiments were conducted in vitro, which cannot fully replicate the complex immune, vascular, and neural microenvironment in vivo. Second, although the upregulation of neurotrophic factors suggests potential for nerve repair, it remains unclear whether functional nerve fibers can grow into implants in vivo. Future studies should: (1) validate the effects of TNT-GO effects on nerve fiber ingrowth and osseoperception recovery in animal models; (2) explore its roles in immune regulation and angiogenesis; and (3) combine TNT-GO with nanodelivery systems to load bioactive molecules or neurotrophic factors to constructing multifunctional implants, improving clinical applicability.

## 5. Conclusions

This study demonstrates for the first time that GO-modified TNT coatings can significantly promote Schwann cell adhesion, proliferation, and neurotrophic factor secretion, providing a new strategy for functional surface modification of implants. Future studies should further validate its effects on nerve regeneration and osseoperception in preclinical models and explore the potential molecular mechanisms and clinical translational potential.

## Figures and Tables

**Table 1:** Primer pairs used in RT-PCR analysis.

Gene	Forward (F)	Reverse (R)
GAPDH	GGCACAGTCAAGGCTGAGAATG	ATGGTGGTGAAGACGCCAGTA
NGF	TGCTGGGCGAGGTGAACATTAAC	GTGTGAGTCGTGGTGCAGTATGAG
GDNF	ACCAAGAAGGCAGAGGCAGAGG	AGACGGCTGTTCTCACTCCTATCC

## Data Availability

The original contributions presented in this study are included in the article/supplementary material. Further inquiries can be directed to the corresponding authors.

## Supplementary Materials

The following supporting information can be downloaded at: https://www.mdpi.com/article/10.3390/jfb17050235/s1, Figure S1: High-magnification SEM images of the Ti, TNT, and TNT-GO surfaces and GO coverage analysis of the TNT-GO surface; Figure S2: Quantitative analysis of Schwann cell live cell ratio on different surfaces at 24 and 48 h.

## Abbreviations

The following abbreviations are used in this manuscript:

TNT-GO	graphene oxide-modified titanium dioxide nanotubes
TNTs	Titanium dioxide nanotubes
GO	graphene oxide
CCK-8	cell counting kit-8
SEM	scanning electron microscopy
AFM	atomic force microscopy
XRD	X-ray diffraction
NGF	nerve growth factor
GDNF	glial cell line-derived neurotrophic factor
DMEM	Dulbecco’s Modified Eagle Medium
FBS	fetal bovine serum
PBS	phosphate-buffered saline
ECM	extracellular matrix
WCA	water contact angle
