# Supplementary material for: Graphene Oxide-Modified Titanium Dioxide Nanotubes Promote Schwann Cell Function and Neurotrophic Factor Expression
Source: J Funct Biomater. 2026 May 8;17(5):235. doi: 10.3390/jfb17050235 (PMC13207634; doi:10.3390/jfb17050235)
Supplement: Supplementary file 1 [file jfb-17-00235-s001.zip › jfb-4276893-supplementary.pdf]

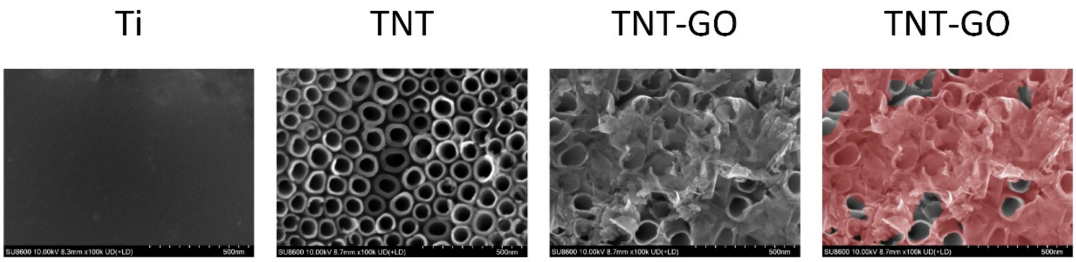

Figure S1. High-magnification SEM images of the Ti, TNT, and TNT-GO surfaces and GO coverage analysis of the TNT-GO surface.

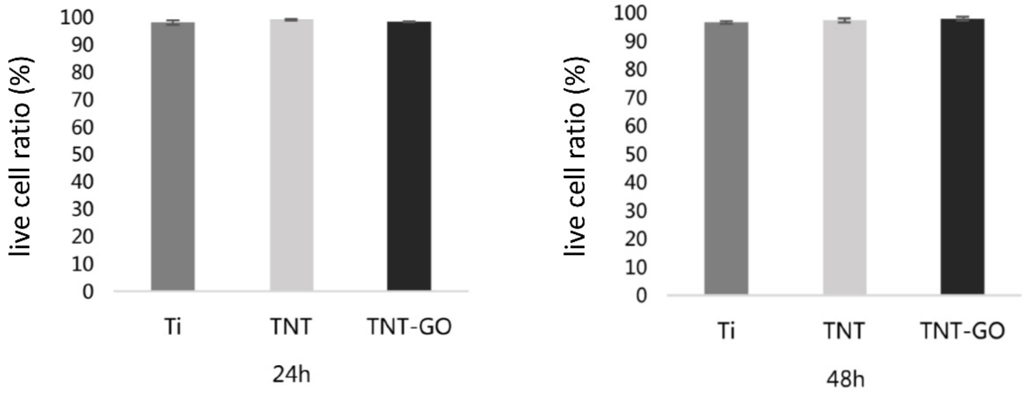

Figure S2. Quantitative analysis of Schwann cell live cell ratio on different surfaces at 24 and 48 h.
